# Neurotoxic effects of AZT on developing and adult neurogenesis

**DOI:** 10.3389/fnins.2015.00093

**Published:** 2015-03-20

**Authors:** Meryem Demir, Eric D. Laywell

**Affiliations:** ^1^Department of Anatomy and Cell Biology, College of Medicine, University of FloridaGainesville, FL, USA; ^2^Department of Biomedical Sciences, College of Medicine, Florida State UniversityTallahassee, FL, USA

**Keywords:** AZT, neurogenesis, subependymal zone, dentate gyrus, thymidine analogs, senescence

## Abstract

Azidothymidine (AZT) is a synthetic, chain-terminating nucleoside analog used to treat HIV-1 infection. While AZT is not actively transported across the blood brain barrier, it does accumulate at high levels in cerebrospinal fluid, and subsequently diffuses into the overlying parenchyma. Due to the close anatomical proximity of the neurogenic niches to the ventricular system, we hypothesize that diffusion from CSF exposes neural stem/progenitor cells and their progeny to biologically relevant levels of AZT sufficient to perturb normal cell functions. We employed *in vitro* and *in vivo* models of mouse neurogenesis in order to assess the effects of AZT on developing and adult neurogenesis. Using *in vitro* assays we show that AZT reduces the population expansion potential of neural stem/progenitor cells by inducing senescence. Additionally, in a model of *in vitro* neurogenesis AZT severely attenuates neuroblast production. These effects are mirrored *in vivo* by clinically-relevant animal models. We show that *in utero* AZT exposure perturbs both population expansion and neurogenesis among neural stem/progenitor cells. Additionally, a short-term AZT regimen in adult mice suppresses subependymal zone neurogenesis. These data reveal novel negative effects of AZT on neural stem cell biology. Given that the sequelae of HIV infection often include neurologic deficits—subsumed under AIDS Dementia Complex (Brew, 1999)—it is important to determine to what extent AZT negatively affects neurological function in ways that contribute to, or exacerbate, ADC in order to avoid attributing iatrogenic drug effects to the underlying disease process, and thereby skewing the risk/benefit analysis of AZT therapy.

## Introduction

AZT (3′-azido-3′-deoxythymidine; AZT, zidovudine or Retrovir) is a synthetic, chain-terminating thymidine analog in which the 3′-hydroxyl group is replaced with an azido group. AZT is converted intracellularly into active AZT-triphosphate (AZT-PPP) which competes with the natural substrate—deoxythymidine triphosphate (dTTP)—for incorporation into elongating viral DNA chains by HIV reverse transcriptase (RT). Once added to a DNA chain, AZT prevents further addition of nucleotides by impeding 5′-3′ phosphodiester linkages via its 3′-azido group. AZT has been used in the treatment of HIV infection, alone, or in combination with other antiviral agents as a part of Highly Active Antiviral Therapy (HAART). The present recommendations for adult patients call for 500–600 mg AZT/day. This dose is approximately equivalent to 10 mg/kg/day, and results in a steady-state serum AZT concentration of 0.8 μM (Fletcher et al., 2002). AZT monotherapy is also recommended to reduce vertical transmission of HIV-1 from mother to child during pregnancy, parturition, and/or breastfeeding. In a non-breastfeeding population AZT monotherapy has been shown to reduce mother-to-child HIV transmission rate from 25.5 to 8.3% (Connor et al., 1994). On the other hand, AZT is still classified in Pregnancy Category C by the Food and Drug Administration due to the potential risks to the fetus (Walker et al., 2007; Durand-Gasselin et al., 2008; Read et al., 2008; Foster et al., 2009).

Although AZT is considered to be effective in reducing viral load, numerous studies show significant adverse side-effects such as bone morrow suppression, cardiomyopathy, hepatotoxicity, neuropathy, and mitochondrial damage (Ayers et al., 1996; Chow et al., 1997; Zhang et al., 1998; Diwan et al., 1999; Anderson et al., 2003; Lee et al., 2003; Lewis, 2003; Lewis et al., 2003, 2004; Lai et al., 2004; Torres et al., 2007). Indeed, abnormal mitochondrial respiratory chain complex activity, alterations in brain morphology, neurological anomalies, cognitive impairments, and episodes of seizures in children exposed to AZT *in utero* and after birth is reported (Blanche et al., 1999, 2006). A greater than expected proportion of birth defects was shown in the central nervous system (CNS), heart, and chromosomes of children exposed to AZT *in utero* (Newschaffer et al., 2000). Congenital CNS anomalies such as anencephaly, microencephaly, and corpus callosum agenesis were reported following prenatal AZT administration (Joao et al., 2010).

Experimental animal models of *in utero* AZT treatment support the clinical human findings. AZT was detected in DNA of fetal liver, lung, heart, skeletal muscle, brain, testis, and placenta in *Macaca mulatta* exposed to AZT during gestation (Poirier et al., 1999; Slikker et al., 2000). Alterations of oxidative phosphorylation complexes were shown in mitochondria of *Erythrocebus patas* brain, heart, and muscle (Ewings et al., 2000; Gerschenson and Poirier, 2000; Gerschenson et al., 2000), and DNA attrition was shown in both monkeys and mice exposed to the drug *in utero* (Olivero et al., 1997). Furthermore, telomeric shortening was observed in a variety of tissues, including the brain, lung, and liver of transplacentally-treated mice (Olivero, 2007). Offspring of AZT-treated rodents were shown to have neurobehavioral abnormalities, suggesting that AZT perturbs CNS development; these abnormalities include deficits in: motor responses; investigative, exploratory, and social behavior; and learning and spatial tasks (Petyko et al., 1997; Busidan and Dow-Edwards, 1999; Calamandrei et al., 1999a,b, 2002a,b; Rondinini et al., 1999; Venerosi et al., 2000, 2003, 2005; Melnick et al., 2005). Especially relevant to our present study, Busidan et al. (2001) have shown that a single dose of 150 mg/kg AZT delivered to pregnant rats on gestational day 20 leads to a heterogeneous distribution of AZT in fetal brains with relatively greater amounts present in the periventricular area. Despite the extensive literature pertaining to AZT toxicity in a variety of systems and cell types, surprisingly little attention has been paid to the potential deleterious effects of AZT administration on neurogenesis.

In mammals, neural stem cells continue to produce new neurons throughout life. Specifically, the subgranular zone (SGZ) of the dentate gyrus and the forebrain subependymal zone (SEZ) are regions where new neurons are produced for the hippocampus and olfactory bulb (OB), respectively. SEZ neural stem/ progenitor cells are located immediately subjacent to the ependymal lining of the anterolateral wall of the lateral ventricle, and there is evidence that stem cells in this region have a ciliated endfoot that intercalates among the ependymal cells to make direct contact with cerebrospinal fluid (CSF) in the ventricle (Mirzadeh et al., 2008; Tavazoie et al., 2008). Such an anatomical arrangement makes it extremely likely that passive diffusion of AZT from the CSF will bring the drug into contact with both SEZ stem cells, and their more fate-restricted progeny that maintain an intimate relationship with the anterior extension of the lateral ventricle as they migrate through the rostral migratory stream to the OB. Likewise, the mouse dorsal hippocampus sits essentially within the posteromedial aspect of the lateral ventricle, and also forms part of the posterosuperior border of the third ventricle. The dentate gyrus itself is located fairly superficially, again making it likely that AZT will diffuse to the site of hippocampal neurogenesis in the SGZ.

The *in vivo* neural stem cell has an *in vitro* correlate known as the neurosphere. Neurospheres are multipotent clones derived from single neural stem cells. Neurospheres consist of neural stem cells that undergo a few rounds of symmetric division, and more fate-restricted progenitors that result from extensive asymmetric division of the stem cells (Deleyrolle and Reynolds, 2009). Furthermore, neurosphere abundance, or yield, *in vitro* has been shown to mirror increases and decreases in levels of *in vivo* neurogenesis (Doetsch et al., 1999). Finally, neural stem/progenitor cells (NSPCs) within the rodent SEZ and hippocampus have been shown to maintain relatively high levels of telomerase activity throughout life (Caporaso et al., 2003), perhaps rendering them uniquely susceptible to AZT toxicity. In this study, we aimed to determine whether clinically relevant AZT regimens perturb normal functioning of NSPCs in developing and adult mouse brain. We hypothesize that the relatively superficial location of NSPC niches of both SGZ and SEZ subjacent to the ventricular lining- will expose these germinal matrices to harmful levels of AZT from the CSF.

## Materials and methods

### Animals

C57BL/6 mice were used for all *in vivo* model systems. Four month old males were used for studies of adult neurogenesis. *In utero* studies were performed on 4 month old pregnant dams, and male and female pups were pooled for analysis. All animals were housed at the University of Florida's Department of Animal Care Services in compliance with Institutional Animal Care and Use Committee (IACUC) regulations.

### Generation and expansion of multipotent astrocyte stem cell (MASC) cultures

Multipotent astrocytic stem cell (MASC) cultures were generated as described (Laywell et al., 2000; Marshall et al., 2008). Briefly, mouse pups were decapitated under hypothermic anesthesia according to institutional veterinary protocol. SEZ tissue surrounding the lateral ventricles was dissected using a sterile razor blade, and the tissue was minced and placed in ice-cold DMEM/F12 medium with N2 supplements (Gibco BRL, 17502-048), (N2 Media), containing 1X antibiotic-antimycotic (Invitrogen, 15240-062) for 15 min. After centrifugation at 400 × g for 5 min, the tissue was incubated in 0.25% Trypsin/EDTA solution (Atlanta Biologicals; B81310) for 5–7 min at 37°C. Trypsin activity was inhibited by adding a bolus of N2 media containing 5% fetal bovine serum (FBS; Atlanta Biologicals). The tissue was triturated into a single-cell slurry using fire-polished Pasteur pipettes of sequentially smaller diameter. Cells were washed in N2 media and pelleted by centrifugation at 400 × g for 5 min before being re-suspended in neural growth medium consisting of N2 medium, 5% FBS, 20 ng/mL recombinant human epidermal growth factor (rhEGF, Sigma-Aldrich, St. Louis, MO; E9644), and 10 ng/mL fibroblast growth factor 2 (bFGF, Sigma-Aldrich, F0291). The single-cell suspension was then plated onto tissue culture flasks (T25) and incubated at 37°C in 5% CO2. After two days of incubation, the neural growth medium was refreshed, and cells were supplemented every other day with EGF and FGF (10 and 20 ng/mL final concentration, respectively) until the primary culture reached confluence.

### Inducible neurogenesis

Confluent primary MASCs were trypsinized and passaged at a density of 17,500 cells/cm^2^ neural growth medium and supplemented as above until confluency was established (typically 7–10 days). To induce differentiation, passage 1 MASC were plated (17,500 cells/cm^2^) onto glass coverslips coated with poly-L-ornithine (10 μg/mL, Sigma, P4957), and were supplemented every other day with 20 ng/mL EGF and 10 ng/mL bFGF. Four days after plating the growth medium was replaced with serum- and growth factor-free N2 medium. This withdrawal of serum and growth factors induces a burst of neuroblast production from the MASC culture. Forty eight hours after withdrawal of neural growth medium, the cells were either fixed with 4% paraformaldehyde in PBS for subsequent immunocytochemical analysis, or were trypsinized and quantified with a Z2 Coulter Counter (Beckman Coulter, Fullerton, CA).

### Immunocytochemistry

Paraformaldehyde-fixed cells were prepared for immunocytochemistry by washing with phosphate buffered saline (PBS) and blocking at room temperature (RT) for 30–60 min in PBS containing 0.01% Triton X-100 (PBSt) and 10% FBS. Primary antibodies were applied overnight in PBSt containing 10% FBS with moderate agitation at 4°C. Residual primary antibody was removed by washing with PBS twice, and secondary antibodies were applied at RT for 1 h in PBSt containing 10% FBS. Residual secondary antibodies were removed by washing with PBS. For nuclear counterstaining, the coverslips were mounted onto glass slides and layered with Vectashield mounting medium containing 4′, 6-diamidino-2-phenylindole (DAPI; Vector, H-1200). Cells were analyzed and photographed by using a Leica DMLB upright epifluorescence microscope (Leica Microsystems AG, Wetzlar, Germany) with a Spot RT color CCD camera (Diagnostic Instruments). For quantification of stained cells, a minimum of 10 randomized fields were selected at 20X magnification.

### Neurosphere culture

Neurospheres (NS) were generated according to methods previously described by our laboratory (Laywell et al., 2002; Marshall et al., 2006). Briefly, single-cell dissociates of neonatal SEZ (as described above) were plated under anchorage withdrawal in low attachment flasks (Nalge Nunc International, 136196) at a density of 10,000 cells/mL. The medium consisted of NeuroCult® NSC Proliferation Medium (Mouse) consisting of NeuroCult® NSC Basal Medium and NeuroCult® NSC Proliferation Supplement (Stem Cell Technologies, 05700 and 05701) supplemented with EGF (20 μg/mL), FGF (10 μg/mL), and heparin (2 μg/mL, Stem Cell Technologies, 07980). Cultures were incubated at 37°C in 5% CO2 for 7 days, at which time the number and diameter of NS were assessed and classified using Spot Advanced digital capture software.

### Neural colony forming cell (NCFC) assay

The neural colony forming cell assay was performed as described by Azari et al. (2011). A single-cell suspension from neonatal SEZ was plated in 35 mm culture dishes at 250 cells/cm^2^ in a serum-free, semi-solid collagen media containing NeuroCult NCFC serum-free medium without cytokines (Stem Cell Technologies, 05720), NeuroCult Proliferation Supplement, hEGF (20 μg/mL), hbFGF (10 μg/mL), and heparin (2 μg/l) for 3 weeks at 37°C in 5% CO2. Cultures were supplemented with Complete Replenishment Medium consisting of NSC Basal Medium, NSC Proliferation Medium, hEGF (20 μg/mL), hbFGF (10 μg/mL), and heparin (2 μg/mL) once a week. Colonies were classified into four categories based on diameter (<0.5 mm, 0.5-mm, 1–2 mm, ≥2 mm) by scanning a gridded scoring dish at 4X magnification.

### Senescence-associated β-galactosidase labeling

X-Gal cytochemical staining at pH 6.0 was performed as described (Dimri et al., 1995). Briefly, cells were fixed for 5 min in 0.2% glutaraldehyde in PBS. After two washes with PBS, cells were incubated in SAβ Gal staining solution containing: 1 mg/mL 5 bromo-4-chloro-3-indolyl B-D galactoside (X-Gal), 40 mM sodium citrate pH 6.0, 5% dimethylformamide, 5% potassium ferrocyanide, 5 mM ferricyanide, 150 mM sodium chloride and 2 mM magnesium chloride for 6 h at 37°C. Cells were washed with PBS, and counterstained with Vectashield + DAPI. The percentage of positive SAβ Gal+ cells was counted on 10 random fields of triplicate samples.

### *In vitro* drug treatment

AZT (TCI America, A2052) was dissolved in N2 medium, filtered through 0.22 μm mesh, and stored in ready-to-use aliquots at −20°C. Based on the literature review, AZT was added to cultures at a final concentration of 0–60 μM, which corresponds to the range of doses administered in human patients (Chiu and Duesberg, 1995). Exposure times ranged from 2 to 48 h, after which the medium was replaced with fresh medium without AZT. Control and treated cultures received the same number of medium changes.

### *In vivo* AZT administration

Presently, the U.S. Food and Drug Administration (USFDA) recommends AZT administration of 600 mg/day, which is about 10 mg/kg for adults. The recommended pediatric dosage is 24–600 mg/day. In order to equate mouse and human doses, we used mg/m^2^ conversion factors based on these USFDA recommendations. Accordingly, a 10 mg/kg dose, which is 600 mg AZT/day, administered to humans with 1.1710 m^2^ body surface area would be equal to a 143 mg/kg dose administered to a mouse with 25 g body weight and 0.007 m^2^ body surface area. Adult, male, C57 BL/6 mice (*n* = 4 per group) received daily i.p. injections of AZT at 0, 1, 10, 20, and 100 mg/kg/day for 2 weeks. On the day following the last AZT injection, all animals received four i.p. injections of the S-phase marker, 5-bromo-3′-deoxyuridine (BrdU;100 mg/kg), every 2 h. Animals were sacrificed 1 week after the final BrdU injection.

### *In utero* AZT administration

C57BL/6 pregnant dams (*n* = 4 per group) received two subcutaneous injections of 200 ul of 0.9% saline containing 0 and 250 mg/kg/day AZT during the last 7 days of gestation (E12-E18; final 37% of gestation period), and for 3 days postpartum in order to expose the pups to AZT via nursing. MASC and neurospheres were generated from the SEZ of litters exposed to AZT.

### Immunohistochemistry

Adult animals were transcardially perfused with 4% paraformaldehyde in PBS. Fixed brains were cryoprotected by immersion in 30% sucrose for 18–24 h. Using a freezing microtome, the hemispheres were cut exhaustively through the sagittal plane at 40 μm, and sections were stored at -20°C in cryoprotectant solution consisting of 50% 0.1 M PO_4_, 25% glycerol, 25% polyethylene glycol. BrdU immunohistochemistry was performed as previously described (Laywell et al., 2005). Briefly, brain sections were washed in PBS and incubated in 2xSSC:formamide (1:1) at 65°C for 2 h. After a wash in 2xSSC, sections were incubated in 2N HCl at 37°C for 30 min. Finally, sections were rinsed in 0.1 M borate buffer at room temperature for 10 min and processed for the standard immunofluorescence detection of BrdU with a rat anti-BrdU antibody (Abcam, Cambridge, MA, ab6326). Sections were co-labeled with an antibody against NeuN, a nuclear antigen specific to mature neurons that delineates the anatomical regions of interest for these studies (i.e., SEZ and hippocampal dentate gyrus). BrdU+ cells were quantified in the SEZ and hippocampal dentate gyrus of each animal using a random 1-in-6 sample of serial sections from the entire left hemisphere. Each series consisted of 17–20 brain sections, depending on total brain size as well as variations inherent to the blocking and sectioning processes. In each section that contained the region of interest, a single focal plane was identified, and only BrdU+ cells within that plane were included for quantification purposes. Due to sectioning thickness and antibody penetration characteristics, the sections used in our analyses contained two distinct focal planes, one of which was excluded in order to avoid potentially counting the same cell twice. The total number of BrdU+ cells present in each sample set was then multiplied by the sampling ratio of 6 to establish an estimate of the total relative number of BrdU+ cells in the region of interest of one hemisphere.

### Statistics

All *in vitro* experiments were conducted in triplicate on identical sister cultures (*N* = 3). *In vivo* experiments were conducted with four animals in each group (*N* = 4). All statistical analyses were performed with GraphPad Prism 4.02 (San Diego, CA). Data were subjected to either One-Way ANOVA with the Tukey–Kramer or Dunnet's Multiple Comparison Test for multiple-group comparisons, or unpaired Student's *T*-test for two-group comparisons. Asterisks (*) indicate groups with statistically significant differences (^*^p < 0.05, ^**^p < 0.01, ^***^p < 0.001).

## Results

### AZT reduces MASC population expansion

**Figure 1:**
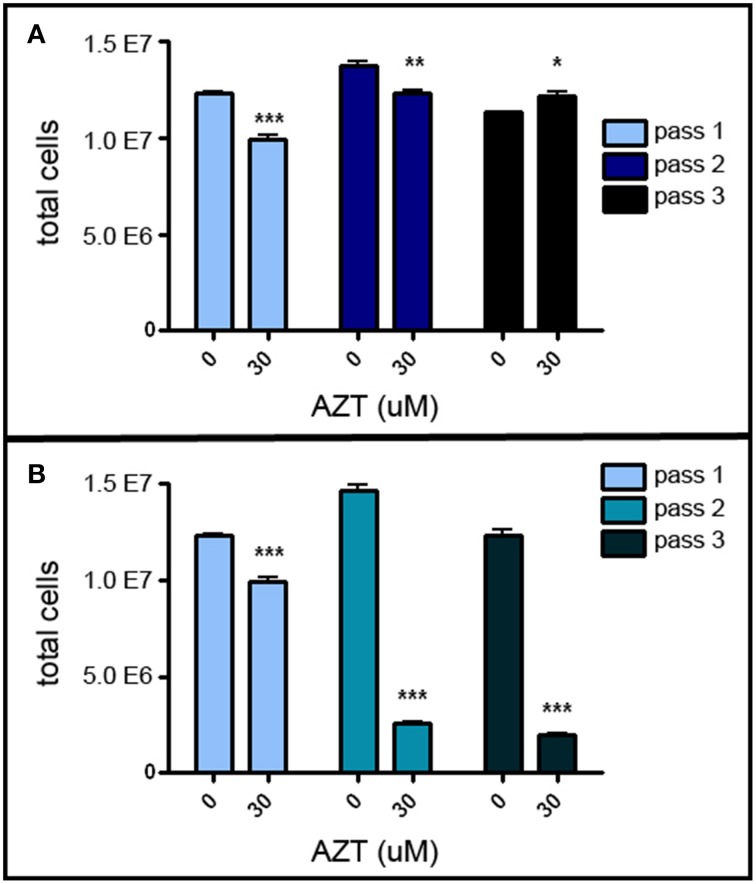
**AZT reduces MASC population expansion. (A)** Primary MASCs exposed to a single 7 day pulse of 30 μM AZT show a significant decrease in population expansion over the first and second passages (1stP, 2ndP). By the third passage (3rdP), treated cells recover and slightly exceed the expansion of untreated cells, although this difference was not maintained, and control and treated groups equilibrated at later passages (not shown). **(B)** Naïve 1stP, 2ndP, and 3rdP MASCs exposed to a single 7 day pulse of 30 μM AZT show that “older” cells (increased in vitro passage) are substantially more vulnerable to the suppressive effects of AZT than “younger” cells. Unpaired *t*-test of significance; *N* = 3 for all groups; ^*^p < 0.05; ^**^p < 0.01; ^***^p < 0.001. Error bars represent standard deviation.

MASC culture enables us to investigate the chain of events involved in proliferation and differentiation of neurogenic stem/progenitor cells. In order to assess the possible adverse effects of AZT on neurogenic stem/progenitor cell expansion, primary MASC were treated with a single-pulse of 30 μM AZT, and growth was analyzed sequentially over three passages. This single-pulse exposure of primary MASC cells to AZT causes a significant decrease in the expansion potential of the progeny over the next two passages (Figure 1). First and second passage AZT-treated cells show a significant reduction in cell numbers compared to their untreated controls (Figure 1A). By the third passage AZT-treated cells have recover and slightly exceed control levels, though control and treated cultures equilibrate at later passages (not shown). These results indicate that the suppressive effect of single-pulse AZT on population expansion is not permanent.

Interestingly, later-passage (“older”) MASC are more susceptible to the suppressive action of single-pulse AZT, and this effect is progressively stronger with increasing *in vitro* “age.” Thirty μM AZT applied at the time of passaging to naïve first, second and third passage MASC causes a more robust suppression of population expansion as compared to application at the time of initial primary cell plating (Figure 1B).

### AZT reduces neurosphere size

**Figure 2:**
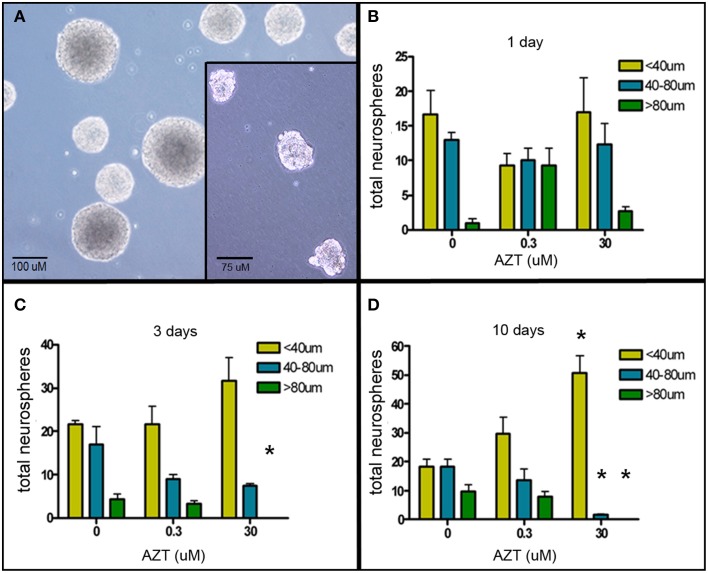
**AZT reduces neurosphere size**. Single-cell dissociates of primary postnatal SEZ form neurospheres within 10 days *in vitro*. AZT (0, 0.3, or 30μM) was added to the culture medium for 1, 3, or 10 days beginning on *in vitro* day 1, and neurospheres were quantified and measured on *in vitro* day 11. **(A)** Representative untreated control neurospheres on day 11. (Inset in **A**) Representative day 11 neurospheres treated with 30 μM AZT for 3 days. AZT reduces neurosphere size in both a concentration- and time-dependent manner **(B–D)**. With increasing exposure time and increasing dose there is a shift in the distribution of neurosphere sizes. While total neurosphere number remains constant among the control and treated groups, AZT induces an increase in smaller neurospheres at the expense of larger neurospheres. Notice the increasing height of the yellow bar (small neurospheres with a diameter less than 40 μM) and a corresponding decrease in the height of the blue and green bars (medium and large neurospheres, respectively). The cultures treated with 0.3 μM AZT show a trend toward smaller neurospheres that does not reach statistical significance compared to untreated controls. Cultures treated with 30 μM AZT show clearly significant loss of medium and large neurospheres at days 3 and 10. One-Way ANOVA, Dunnett's Multiple Comparison Test of significance; *N* = 3 for all groups; ^*^p < 0.05. Asterisks indicate a significant difference as compared to the corresponding size range of neurospheres in matched, untreated cultures. Error bars represent standard deviation.

The neurosphere (NS) assay is a second cell culture system that allows us to investigate the effects of AZT on neurogenic stem and progenitor cells (Deleyrolle and Reynolds, 2009). NS-forming cells were treated the day after plating with a single pulse of AZT at either 0.3 or 30 μM. AZT remained in the culture medium for 1, 3, or 10 days (Figure 2). Quantification of NS number and size on day 10 reveals that there is no difference in total NS number between untreated controls and either of the AZT-treated groups; however, AZT does cause a significant reduction in neurosphere size in both a time- and concentration-dependent manner as compared to untreated NS-forming cell controls (Figures 2B–D). While total NS number among the groups does not differ, there is a progressive reduction in the number of large NS (>80 μM diameter, green bars), and a corresponding increase in the number of small NS (<40 μM diameter, yellow bars) and medium NS (40–80 μM diameter, blue bars) in cultures treated with AZT. This effect is stronger in the cultures receiving 30 μM AZT, and becomes more pronounced with increasing AZT application time (i.e., notice the emergence of exclusively small neurospheres in the 10 day), 30 μM AZT cultures (Figure 2D). The fact that NS frequency is not altered suggests that AZT does not directly interfere with the survival of NS-forming cells. However, NS diameter is used as a metric of the capacity for self-renewal (Marshall et al., 2007), and the clear reduction in NS size suggests an AZT-mediated perturbation of normal proliferative capacity.

### AZT perturbs neural colony formation by stem and progenitor cells

**Figure 3:**
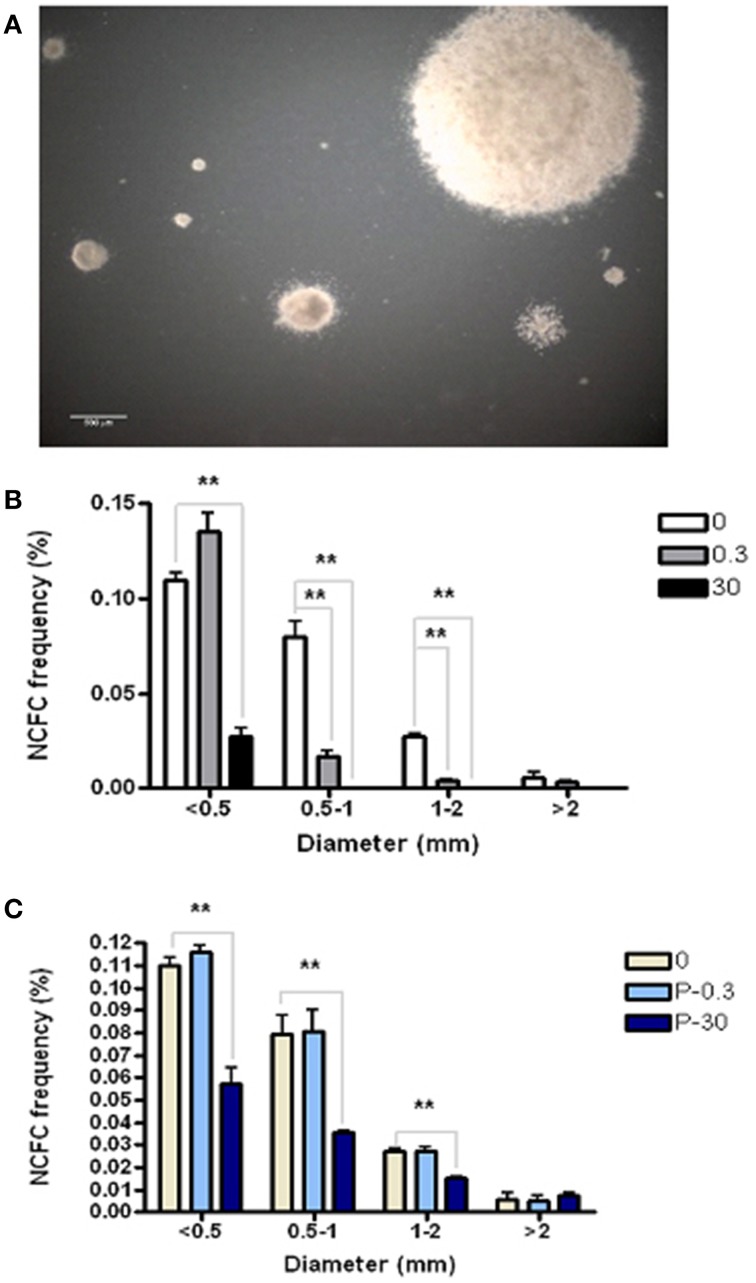
**AZT perturbs neural colony formation by both neural stem cells and neural progenitor cells. (A)** Dissociated primary neurosphere cells were cultured in the Neural Colony-Forming Cell Assay in the presence of 0.3 or 30 μM AZT. After 21 days *in vitro*, colonies were classified into one of four categories based upon diameter. **(B)** AZT at both concentrations causes a decrease in the size of neural colony formation by both stem cells (≥2 mm) and progenitor cells (<2 mm). **(C)** Pre-treatment of primary NS with 0.3 or 30 μM AZT prior to dissociation into the NCFC Assay does not affect colony formation as severely as direct AZT application after dissociation. Here, 30 μM AZT pre-treatment disturbs the formation of colonies derived from neural progenitor cells (<2 mm), but does not decrease colonies derived from stem cells (≥2 mm). P, pre-treatment; One-Way ANOVA, Dunnett's Multiple Comparison Test of significance; *N* = 3 for all groups; ^**^p < 0.001. Error bars represent standard deviation. NCFC frequency (%) = (number of colonies/total cells plated) (100).

The Neural Colony Forming Cell (NCFC) assay allows us to distinguish between and quantify neural stem cell (NSC) vs. neural progenitor cell (NPC) frequency. NSCs, having higher proliferative potential, form colonies ≥2 mm in diameter in semi-solid medium. On the other hand, more restricted neural progenitor cells (NPC) lack the extensive self-renewal of NSC, and therefore form colonies that are always <2 mm in diameter under these culture conditions (Deleyrolle et al., 2011). In order to determine if AZT affects the ability of primary NSC and/or NPC to form colonies, we exposed dissociated primary NS (containing a mixture of NSC and NPC) to a single pulse of either 0.3 or 30 μM AZT. After 21 days in culture, colonies were classified into four categories based on diameter (Figure 3). Our data show (Figure 3B) that single-pulse AZT exposure causes a concentration-dependent decrease in the formation of neural colonies derived from both NPC (<2 mm in diameter) and NSC (>2 mm in diameter). In order to further examine potential differential effects of AZT on NSC vs. NPC we exposed primary neurospheres to a single pulse of 0.3 and 30 μM AZT before dissociation and re-plating in the NCFC assay. Our results show that pre-treatment with 30 μM AZT disturbs formation of colonies smaller than 2 mm in diameter, colonies derived from NPCs, but not colonies larger than 2 mm which are colonies derived from NSCs (Figure 3C). This result suggests the NPC may be more at risk for the suppressive effects of AZT, since the “window” of susceptibility to exposure seems to be longer than that for NSC.

### AZT attenuates inducible neurogenesis from MASC

**Figure 4:**
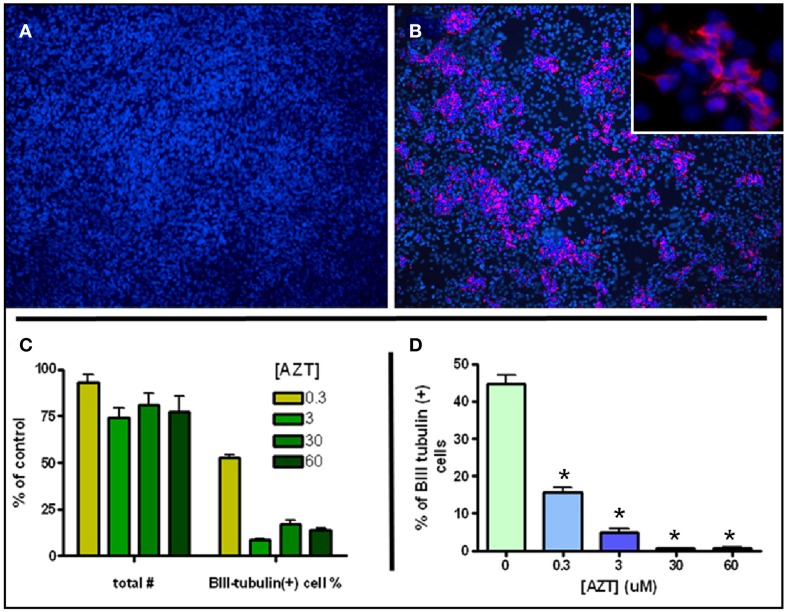
**AZT Attenuates Inducible Neurogenesis from MASC**. Serum and mitogen withdrawal for 48 h induces neurogenesis from MASC cultures. Representative immunofluorescence images show control **(A)** and induced **(B)** MASC cultures immunolabeled for β-III tubulin (red) and DAPI (blue). Inset in **(B)** reveals the morphology of newly-generated, β-III tubulin+ neuroblasts at higher magnification. **(C,D)** A single 48 h-pulse of 0.3, 3, 30, or 60 μM AZT initiated at the time of withdrawal causes a mild decrease in total cell number, but a severe reduction in the number of induced neuroblasts. Treated cultures contain 75–90% as many total cells as untreated controls (panel **C**, left four bars). However, treated cultures contain only 10–50% as many β-III tubulin-positive neuroblasts as control cultures (panel **C**, right four bars). Expressed as a percentage of total cells **(D)**, neuroblasts in untreated induced cultures comprise about 45% of all cells, while in AZT-treated induced cultures neuroblasts represent from 15% (0.3 μM) to less than 2% (30 and 60 μM) of total cells. One-Way ANOVA, Dunnet's Multiple Comparison Test of significance; *N* = 3 for all groups; Asterisk indicates values significantly different from untreated controls, ^*^p < 0.01. Error bars represent standard deviation.

MASC can be induced to generate large numbers of neuroblasts upon withdrawal of serum and mitogens (Scheffler et al., 2005). MASC isolated from SEZ were grown to confluence on an adhesive surface in the presence of serum and the mitogens EGF and bFGF. Twenty four hours after withdrawal of serum and mitogens, MASC generate characteristic rosettes of cell clusters consisting of B-III-tubulin+ neuroblasts (Figures 4A,B). To assess the effect of AZT on this model of inducible neurogenesis, we exposed MASC to AZT at the time of induction (serum and mitogen withdrawal). We show that a single 48 h-pulse of AZT (0.3–60 μM) at the time of withdrawal causes a mild decrease in total cell number, but a severe reduction in neuroblast induction (Figures 4C,D). Total cell number was reduced approximately 10–25% in the treated cultures, while neuroblast induction was reduced by 50–90% as compared to untreated controls.

**Figure 5:**
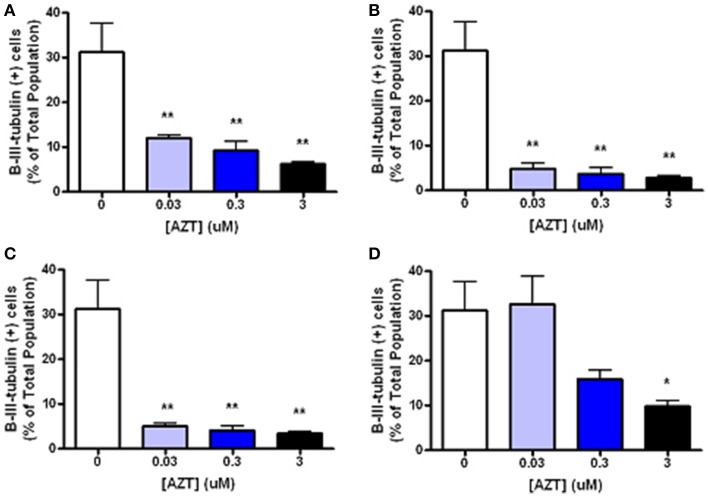
**Short, low-dose AZT exposure at the time of withdrawal is sufficient to perturb inducible neurogenesis**. At the initiation of serum and mitogen withdrawal MASCs were treated with a single pulse of 0.03, 0.3, or 3 μM AZT for 2, 8, or 24 h (**A**–**C**, respectively). All AZT doses suppress neuroblast induction in a dose- and time-dependent manner. Even the lowest concentration of AZT, 0.03 μM, applied for only 2 h at the time of serum and mitogen withdrawal significantly decreases neuroblast formation compared to the control group. In contrast, AZT pre-treatment with the same concentration range for 3 days prior to supplement withdrawal **(D)** is less disruptive of neurogenesis; there is a dose-related trend in suppressed neuroblast formation, but only the highest dose (3 μM) causes a statistically significant reduction. One-Way ANOVA, Dunnet's Multiple Comparison Test of significance; *N* = 3 for all groups; ^*^p < 0.05; ^**^p < 0.01. Error bars represent standard deviation.

In order to examine the minimum duration and dosage of AZT exposure required to perturb inducible neurogenesis, MASC were treated with for 2, 8, or 24 h with AZT (0.03–3 μM) at the time of serum and mitogen withdrawal (Figure 5). We show that even a 2 h exposure to the lowest concentration of AZT significantly suppresses neuroblast formation (Figures 5A–C). On the other hand, MASC treated with the same concentration range of AZT for 3 days prior to supplement withdrawal do not show a much less dramatic response (Figure 5D), suggesting that AZT must be present at the time of induction in order to exert an inhibitory effect upon neuroblast generation.

### AZT induces a senescence phenotype in MASC

**Figure 6:**
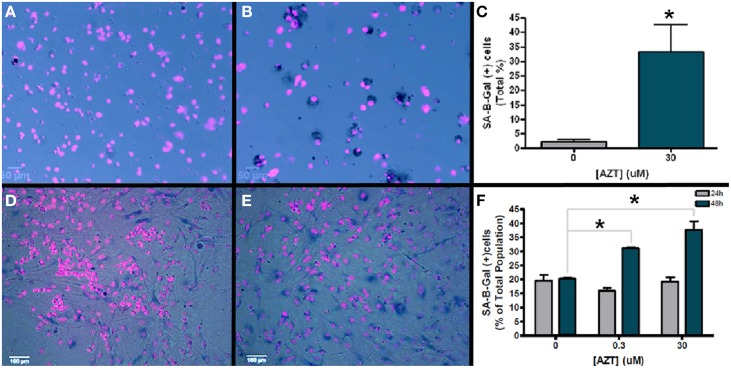
**AZT-treated cells upregulate senescence-associated beta galactosidase (SA-B-Gal)**. Neurosphere-forming cells were treated with a single pulse of AZT (30 μM) 24 h after plating. Subsequent neurospheres were dissociated and the cells were processed for SA-B-Gal histochemistry. **(A)** Untreated control neurosphere cells show low levels of SA-B-Gal activity as compared to AZT-treated neurosphere cells **(B)**. Graphical representation of the quantitative results **(C)** shows that SA-B-Gal labeling is approximately 15-fold higher in AZT-treated cells (2% of total control cells vs. 33% of total treated cells; unpaired *t*-test, *p* < 0.05). Similar results were obtained with MASC exposed to a single 48 h. pulse of either 0.3 or 30 μM AZT. In this case, untreated control astrocytes **(D)** show a higher baseline level of SA-B-Gal labeling than dissociated neurospheres, but the AZT-treated astrocytes **(E)** show a clear and substantial upregulation of this senescence marker. Graphical data for astroctyes **(F)** shows that 0.3 and 30 μM AZT for 48 h. (green bars) cause a respective 50 and 75% increase in SA-B-Gal+ cells (One-Way ANOVA, Dunnett's multiple comparison test of significance, *p* < 0.01). However, 24 h. exposure (gray bars) to these concentrations does not significantly alter SA-B-Gal expression. Asterisks indicate values significantly different from control; error bars represent standard deviation. *N* = 3 for all groups; error bars represent standard deviation.

Senescence associated B-galactosidase (SA-B-Gal) activity has been used as a biomarker to detect senescent cells *in vitro* and *in vivo* (Dimri et al., 1995). In order to determine if AZT causes an increase in senescence associated B-galactosidase activity, we exposed neurosphere-forming cells to a single pulse of 30 μM AZT 24 h after plating. Subsequently, neurospheres formed from these treated cells were dissociated and assayed for SA-B-Gal expression (Figures 6A,B). Our results show an approximate 15-fold increase in SA-B-Gal expression in the AZT-treated neurosphere cells as compared to untreated controls (Figure 6C; 2 vs. 33%).

In addition, we also examined senescence phenotype in AZT-treated MASC. After a single 24 or 48 h pulse of 0.3 or 30 μM AZT MASC were assayed for SA-B-Gal expression (Figures 6D,E). We find that there is no significant difference in SA-B-Gal expression between untreated controls and AZT-treated MASC with either dose after 24 h of exposure (Figure 6F, gray bars). However, at 48 h of exposure, both 0.3 and 30 μM AZT is associated with upregulation of SA-B-Gal as compared to untreated controls (Figure 6F, green bars).

### *In vivo* AZT administration reduces proliferation in adult germinal zones

**Figure 7:**
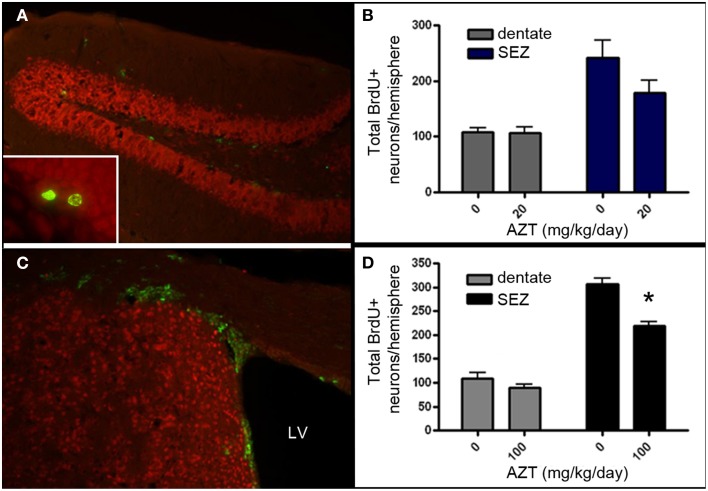
**AZT reduces SEZ but not hippocampal neurogenesis *in vivo***. Adult mice received a 2-week regimen of AZT (20 or 100 mg/kg/day, i.p.). On the day after the last AZT injection, animals received 4 successive injections of BrdU (100mg/kg) at 2-h intervals. One week later the animals were euthanized and the brains were processed and analyzed. Representative epifluorescence images of coronal sections through the hippocampal dentate gyrus **(A)** and SEZ **(C)** of the adult mouse immunolabeled for BrdU (green) and NeuN (red). Inset in **(A)** shows two BrdU+ (green) cells in the molecular layer of the dentate gyrus, immediately superficial to the subgranular zone.Neither the 20 mg/kg/day **(B)** nor the 100 mg/kg/day **(D)** regimen significantly altered the number of newly-generated cells (green) in the dentate gyrus (gray bars in both **B,D**). However, both regimens reduced proliferation in the SEZ (blue bars in **B**, black bars in **D**), though only the 100 mg/kg AZT dose reached statistical significance. Unpaired Student's *t*-test. NS, no significant difference. Asterisk indicates significant difference, *p* < 0.01. *N* = 4 for all groups. Error bars represent standard deviation.

Given that AZT is classified as a Rank 1 drug with respect to CNS penetration, we aimed to determine if *in vivo* administration of AZT alters the proliferation potential of NSPCs within the persistent neurogenic niches of the vertebrate brain. We administered AZT to adult male mice at 0, 20, or 100 mg/kg/day via daily intraperitoneal injection for 2 weeks. On the day following the last AZT injection, all animals received 4 BrdU injections (100 mg/kg) at 2 h intervals. After an additional week the animals were sacrificed and their SEZ and dentate gyrus were processed for BrdU immunohistochemistry (Figures 7A,B). Our results show that 2 week administration of low dose AZT (20 mg/kg/day) does not alter the number of BrdU+ cells present in either the dentate gyrus or the SEZ (Figure 7C). However, the moderate dose of 100 mg/kg/day is associated with reduced numbers of BrdU+ cells in both the dentate gyrus and the SEZ, although only the difference for the SEZ reaches statistical significance compared to controls (Figure 7D).

### *In utero* AZT administration perturbs NSPC proliferation and neurogenesis

**Figure 8:**
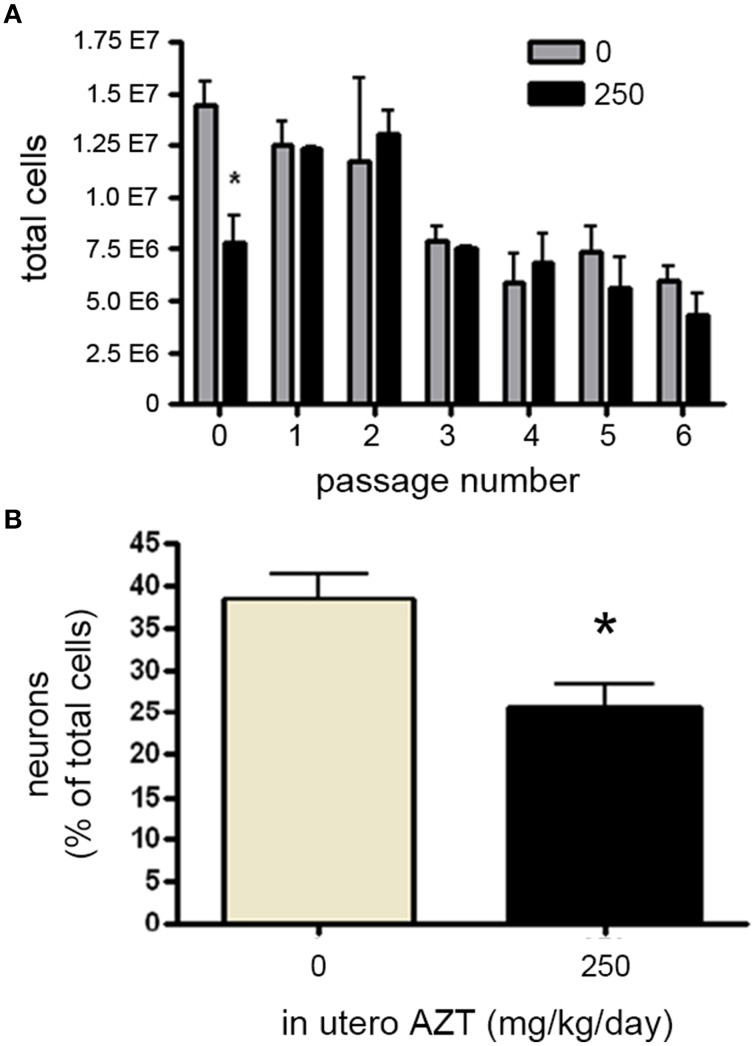
***In utero* AZT perturbs MASC proliferation and differentiation**. AZT was administered to pregnant dams from day 12 of gestation until postnatal day 3 (250 mg/kg/day, s.q.). At postnatal day 3 primary MASC were generated from the SEZ of the pups. The MASC were assayed for inducible neurogenesis at passage 1, and were assayed for population expansion over 6 passages. **(A)** *In utero* AZT administration also decreases the expansion potential of primary MASC, but this deficit is not maintained over 6 passages as control and treated MASC equilibrate in population expansion after passaging. **(B)** Inducible neurogenesis in MASC cultures derived from pups exposed to AZT *in utero* is reduced by approximately 35% compared to age-matched pups born to untreated mothers. Unpaired Student's *t*-test; *N* = 3 for all groups; ^*^p < 0.05.Error bars represent standard deviation.

We next investigated whether *in utero* exposure to AZT perturbs prenatal and early postnatal neurogenesis, AZT (250 mg/kg/day s.q.) was administered to pregnant females/dams between day 12 of gestation and postnatal day 3 (P3). This dosing regimen was chosen to correspond to the current clinical approach to treating pregnant, HIV-positive women in an attempt to prevent vertical (mother-to-child) transmission of infection. We found no significant differences in litter size, pup weight, or weight of the dams between control and AZT-treated groups (data not shown). In order to assess the effect of *in utero* AZT on neurogenic stem/progenitor cell expansion, primary MASC were generated from the offspring on P3. Our results show that *in utero* AZT administration is associated with decreased expansion potential of primary (passage 0) MASC derived from offspring SEZ (Figure 8A). As can be seen from this graphical representation, AZT-treated and control MASC equilibrate at passage 1, and do not show statistically significant differences in expansion potential through passage 6, the last passage analyzed.

We additionally examined the effect of *in utero* AZT on inducible neurogenesis from MASC. Passage 2 MASC derived from P3 treated and control SEZ were induced by withdrawal of serum and growth factors. Forty-eight hours later cells were fixed and immunolabeled for B-III-tubulin+ neuroblasts. We find that *in utero* AZT significantly decreases the inducible neurogenesis potential of MASC cells (Figure 8B). The data show an approximate 30% reduction in mean neuroblast production in AZT-treated animals as compared to untreated controls (compare tan and black bars in 8 B).

**Figure 9:**
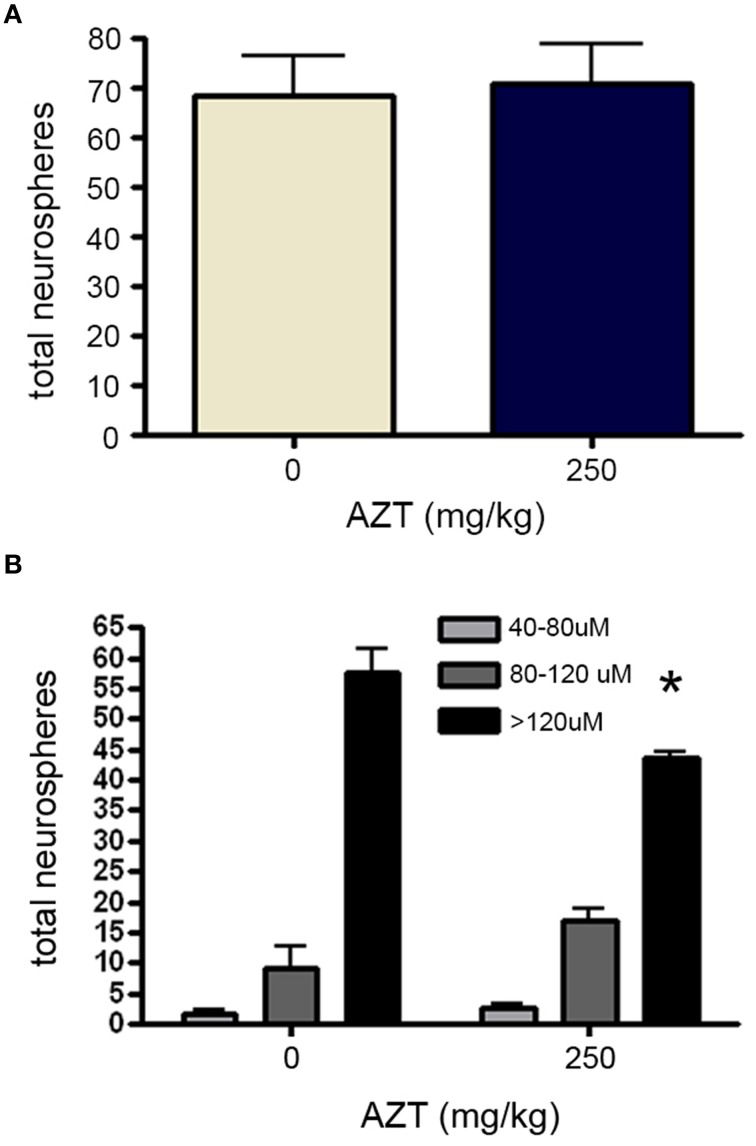
***In utero* AZT reduces neurosphere size**. AZT was administered to pregnant dams from day 12 of gestation until postnatal day 3 (250 mg/kg/day, s.q.). Single-cell dissociates of SEZ were prepared from pup brains on postnatal day 3 and assayed for neurosphere production. **(A)** The total number of neurospheres generated from AZT-treated vs.control pups does not differ. **(B)** While total neuroshere number does not difffer between treated and control groups, *In utero* AZT causes a clear and significant reduction in the diameter of neurospheres generated from the pups of treated mothers as compared to the pups of untreated control mothers; notice the reduction in the the number of large diameter neurospheres (>120 μM) and a corresponding increase in the number of small and medium diameter neurospheres (40–120 μM) in the the treated group as compared to the controls. Unpaired Student's *t*-test for **(A)**; One-Way ANOVA for **(B)** *N* = 3 for all groups; ^*^p < 0.05. Error bars represent standard deviation.

Finally, in order to determine if *in utero* AZT exposure alters the growth potential of NS-forming cell progeny, we isolated and plated primary SEZ dissociates from the offspring at P3. After culturing for 10 days NS were quantified and measured. Our results show that NS frequency does not differ between AZT-treated pups and untreated control pups (Figure 9A). However, as we saw with NS-forming cells treated with AZT *in vitro*, there is a difference in the distribution of NS sizes (Figure 9B). Significantly fewer large diameter NS (>120 μM) are derived from AZT-treated pups, but this is offset by a corresponding increase in small and medium diameter NS (40–120 μM).

## Discussion

We have examined the effect of AZT on the biology of NSCs and NPCs. Using both *in vitro* and *in vivo* models we found that AZT administration is associated with severe perturbations in both proliferative capacity and neurogenesis. These findings are somewhat surprising, given the general understanding of AZT's limited penetration into the brain parenchyma, and they may have clinical implications pertaining to cognitive deficits in persons receiving antiretroviral therapy. Though AZT has previously been shown to accumulate within the superficial periventricular regions of the brain [40], surprisingly, little attention has been paid to potential AZT toxicities involving NSC and NPCs that have been localized to these areas.

AZT is classified as having a high rank of CNS penetration—effectiveness (CPE) based upon its chemical features and measured CSF concentrations (Wu et al., 1998; Kearney and Aweeka, 1999; Letendre et al., 2008; Im et al., 2009). However, there is evidence showing that AZT is poorly distributed to the brain due to the fact that it is not actively transported across the blood-brain barrier (BBB), but relies on diffusional processes for penetration (Thomas and Segal, 1997). The BBB is formed by cerebral blood vessel endothelial cells in concert with astrocyte endfeet, and creates a barrier between blood and the brain parenchyma. Similarly, the BBB and the BCSFB -formed by blood vessels within choroid plexuses of the lateral, third, and fourth ventricles- play an important role in distribution of AZT to the brain. While passive diffusion of AZT through the BBB and BCSFB is a relatively slow process (Thomas and Segal, 1997; Kearney and Aweeka, 1999; Cysique et al., 2004; Evers et al., 2004; Letendre et al., 2004), AZT is rapidly removed from the brain via an active probenecid-sensitive transport efflux (Dykstra et al., 1993; Takasawa et al., 1997a,b; Sawchuk and Yang, 1999). For these reasons there is significant pessimism regarding the therapeutic efficacy of AZT in the treatment of central manifestations of HIV infection (Groothuis and Levy, 1997).

HIV infection in the CNS leads to the development of asymptomatic neurocognitive impairment, HIV-associated mild neurocognitive disorder (MND), and AIDS dementia complex (ADC) or HIV associated dementia (HAD) with impairment in cognitive activity, memory, attention, and motor and behavioral functioning (Antinori et al., 2007). Therefore prevention of HIV infection in the CNS is a major clinical goal. In order to enhance levels of antiretroviral drugs including AZT in CNS and to make them more efficient, researchers focus on developing new strategies such as intranasal administration of AZT, developing BBB-permeable derivatives of antiretroviral drugs and efflux inhibitors, and modulating transporters (Zhikova and Stankova, 2000; Eilers et al., 2008; Miller et al., 2008; Quevedo et al., 2008; Im et al., 2009; Li et al., 2010; Saiyed et al., 2010). On the other hand, the potential effects of direct exposure of excessive AZT concentrations and immune response to the toxicity on the CNS are not known. We suggest that in the case of increased long-term delivery of AZT into the brain, a direct exposure to the CNS would cause more dramatic changes than we have shown in our short-term *in vitro* and *in vivo* assays. Indeed, it has been reported that antiretroviral drugs with good CNS penetration are associated with poor neurocognitive performance of advanced HIV patients (Marra et al., 2009). We believe that the superficial location of the neurogenic niches with respect to the ventricular spaces, and the unique cytoarchitecture of the SEZ stem cells, which contact both the lateral ventricle and cerebral blood vessels, exposes neural stem cells to high levels of circulating AZT.

The present results show that AZT reduces the proliferative capacity of exposed cells, both *in vitro* and *in vivo*. Our culture assays of MASC, NS, and NCFC all show attenuated population expansion that is accompanied by a concomitant increase in senescence-associated beta galactosidase activity. Likewise, MASC, and NS derived from the brains of animals exposed *in utero* to AZT show similar impairments in proliferation when compared to untreated controls. In addition to suppressing proliferative capacity, AZT also clearly inhibits the capacity for NSPCs to generate new neuroblasts. *In vitro*, this effect is seen using a model of inducible neurogenesis from MASC cultures derived from the SEZ. This action occurs relatively quickly, as it requires AZT to be present at the initiation of induction. To examine whether *in vivo* exposure of AZT disrupts proliferation within adult neurogenic zones, we injected adult animals with AZT at clinically relevant low (20 mg/kg/day) and moderate (100 mg/kg/day) concentrations for a short time period (2 weeks). Proliferation was assessed by BrdU incorporation within the dentate gyrus and SEZ. These analyses reveal that the low dose does not perturb proliferation in either the dentate gyrus or the SEZ. However, the moderate dose initiated a decline of proliferation in both of the regions. It seems, then that both of these neurogenic niches are potentially susceptible to systemically-administered AZT, and the will likely show more dramatic impairments with a longer treatment regimen that is more similar to the human therapeutic condition. We also examined whether perinatal exposure of AZT perturbs prenatal and early postnatal neurogenesis. Dams were treated with 250 mg/kg/day of subcutaneous AZT from day 12 of gestation until postnatal day 3, mimicking the treatment regimen recommended for pregnant HIV+ women. This treatment paradigm was not overtly toxic, as the litter sizes and pup weights were not significantly different from controls (data not shown). However, inducible neurogenesis was reduced by about 30% in MASC cultures derived from treated pup brains.

The mechanistic causes of the negative sequelae related to AZT exposure are beyond the scomp of our present report. However, there are four recognized, direct mechanisms by which AZT might exert a toxic effect on normal cells, and it is possible that synergistic combinations of these mechanisms account for the clinical side effects of AZT therapy. First, it may be that the original ID50 reports for AZT, obtained with the cancerous H9 T-cell line, do not translate accurately to other cell types. In fact, subsequent investigations have revealed radically lower ID50 values for cell proliferation than were originally described in the study by Furman et al. (1986) suggesting that, at therapeutically prescribed dosages, DNA polymerase α does incorporate triphosphorylated AZT into cellular DNA chains during replication (Inoue et al., 1989; Mansuri et al., 1990). Additionally, while there is a 100-fold selectiveness of AZT for viral RT over DNA polymerase α, the fact that the human genome is approximately 3 × 10^5^ times larger than the HIV genome means that there is still a high probability of AZT inserting into—and terminating—cellular DNA chains during each round of cell division (Chiu and Duesberg, 1995).

Second, while RT has a greater affinity for AZT than do the cellular DNA polymerases responsible for normal chromosomal replication, there are other polymerases within eukaryotic cells that might also incorporate AZT. For instance, polymerase γ (gamma)—the enzyme responsible for the replication and maintenance of the mitochondrial genome—also incorporates nucleoside analogs efficiently (Lee et al., 2003). The pathological effects of such incorporation might include the loss of mitochondrial DNA and/or severe oxidative stress (Lewis, 2003), which would account for some of the symptomology associated with antiretroviral therapy. Indeed, both *in vitro* and *in vivo* studies have consistently revealed deleterious effects of AZT on oxidative phosphorylation (Hobbs et al., 1995) and mtDNA synthesis (Simpson et al., 1989). Perturbations of mitochondrial DNA polymerase γ may underlie such dysfunctions, as shown by Lewis et al. (1994), who demonstrated that AZT-triphosphate (TP) inhibits bovine polymerase γ through both competitive and non-competitive mechanisms.

Third, AZT may perturb the phosphorylation of naturally-occurring, intracellular nucleotide pools. While both the naturally-occurring and synthetic nucleosides must undergo intracellular tri-phosphorylation before they can be incorporated into DNA chains, not all nucleosides are phosphorylated with the same efficiency. There is evidence that *in vivo* tri-phosphorylation of AZT is very inefficient; however, AZT is abundantly mono- and di-phosphorylated, and accumulations of AZT-MP and AZT-DP can perturb normal thymine phosphorylation (see Papadopulos-Eleopulos et al., 1999 for a review). For example, it has been shown that rat cardiac mitochondria, can process AZT only to the monophosphorylated form, and accumulation of AZT-MP acts as a competitive inhibitor of subsequent thymine phosphorylation (McKee et al., 2004). In fact, in the original AZT toxicity study *in vitro* phosphorylation kinetics showed that the presence of AZT leads to a massive intracellular decrease in triphosphorylated forms of thymidine, cytidine, and guanosine, while triphosphorylated adenosine is increased (Nakashima et al., 1986). It is likely that such pathological intracellular processing characteristics of AZT underlie some of the toxic effects of antiretroviral therapy.

Fourth, AZT might exhibit a direct toxic effect through disruption of telomerase activity, leading to shortened telomeres. Telomeres are repeating TTAGGG sequences that cap the ends of chromosomes, and are believed to protect against end-to-end chromosome fusion. Since DNA polymerase cannot completely replicate linear DNA strands, telomeres grow progressively shorter with each cell division in most somatic cells. However, highly proliferative cells such as germline cells, cancer cells, and stem and progenitor cells maintain telomere length during mitosis via the activity of telomerase (Singer and Berg, 1990). Telomerase is a ribonucleoprotein that maintains telomere length by adding telomere repeats to the 3′ end of DNA strands after cell division. This is accomplished by the reverse transcription of its AAUCCC RNA sequences into TTAGGG DNA sequences, and their attachment to the ends of chromosomes. Telomerase, therefore, is a reverse transcriptase that, like HIV RT, may be a target for AZT-mediated chain termination. If this is the case, there is reason to believe that neurogenesis and neural differentiation will be adversely affected. Caporaso et al. (2003) showed *in vivo* that telomerase activity is absent from most of the adult rodent brain, but that high activity is maintained in the germinal niches that are responsible for persistent neurogenesis. *In vitro* studies, too, have shown that AZT-mediated telomerase perturbation leads to reduced proliferation of embryonic mouse cortical neuronal precursor cells (Haik et al., 2000). Finally, knockout mice (*Terc*^−/−^), that are telomerase deficient due to a deletion in the telomerase RNA component, show telomere attrition that is linked to perturbation of both proliferation and differentiation of adult neural stem cells (Ferron et al., 2004, 2009).

Finally, since our results suggest that AZT impairs proliferation, it may be that AZT perturbs growth through the induction of senescence pathways. Recently, we have reported that senescence is induced by the uptake of the non-chain-terminating thymidine analogs, bromodeoxyuridine (BrdU), and ethynyldeoxyuridine (EdU). Specifically, incorporation of these analogs leads to suppressed proliferation among treated cells and their progeny (Levkoff et al., 2008; Ross et al., 2008, 2011). Senescence induction may be a property of the larger family of synthetic thymidine analogs, including AZT. While the mechanism behind thymidine analog-induced senescence has so far remained enigmatic, there are a number of plausible mechanisms that may be involved. These include perturbation of nucleoside phosphorylation kinetics, telomerase inhibition, and mitochondrial dysfunction. Alternatively, an as-yet unidentified mechanism may be responsible for the senescent phenotype. Recent proteomic work by Ukekawa et al. (2007) shows that lamin A/C and pre-lamin A are the most abundantly upregulated proteins in both senescing normal human fibroblasts and HeLa cells that had been exposed to BrdU. Additionally, they found that mRNA coding for Zmpste 24 (FACE-1), the enzyme responsible for proteolytic cleavage of the pre-lamin A CAAX motif, was severely reduced in both senescing fibroblasts and HeLa cells after exposure to BrdU. Since mutation of the lamin A gene has been causally linked to the senescence human phenotype, Hutchinson-Guilford progeria (Eriksson et al., 2003), it seems reasonable to suspect that thymidine analog disruption of the nuclear envelope underlies the senescence of cells exposed to BrdU and AZT.

Together, our data reveal uncharacterized negative consequences of AZT treatment on neural stem and progenitor cells. Most of the toxic effects of AZT in humans occur after long time scales, beyond the capability of our in *in vivo* experiments with short-term treatment models. The long-term use of AZT as a part of anti-HIV therapy seems likely to affect NSPCs within the adult brain. Given the fact that HIV infection leads to development of neurological deficits and that human HIV+ patients are treated with AZT over multiple years, it is important to determine to what extent AZT regimens might perturb normal levels of neurogenesis to exacerbate or contribute to these neurological problems.

### Conflict of interest statement

The authors declare that the research was conducted in the absence of any commercial or financial relationships that could be construed as a potential conflict of interest.
